# Murine CD8 T‐cell functional avidity is stable in vivo but not in vitro: Independence from homologous prime/boost time interval and antigen density

**DOI:** 10.1002/eji.201948355

**Published:** 2019-12-10

**Authors:** Connie B. Gilfillan, Chensu Wang, Mona O. Mohsen, Nathalie Rufer, Michael Hebeisen, Mathilde Allard, Grégory Verdeil, Darrell J. Irvine, Martin F. Bachmann, Daniel E. Speiser

**Affiliations:** ^1^ Department of Oncology University of Lausanne Switzerland; ^2^ Koch Institute for Integrative Cancer Research MIT Cambridge MA USA; ^3^ Inselspital Universitaetsklinik RIA, Immunologie Bern Switzerland; ^4^ Jenner Institute University of Oxford Oxford UK; ^5^ Department of Oncology Lausanne University Hospital Lausanne Switzerland

**Keywords:** Avidity regulation, Functional avidity, Prime/boost, T‐cell receptor affinity, T‐cell vaccination

## Abstract

It is known that for achieving high affinity antibody responses, vaccines must be optimized for antigen dose/density, and the prime/boost interval should be at least 4 weeks. Similar knowledge is lacking for generating high avidity T‐cell responses. The functional avidity (FA) of T cells, describing responsiveness to peptide, is associated with the quality of effector function and the protective capacity in vivo. Despite its importance, the FA is rarely determined in T‐cell vaccination studies. We addressed the question whether different time intervals for short‐term homologous vaccinations impact the FA of CD8 T‐cell responses. Four‐week instead of 2‐week intervals between priming and boosting with potent subunit vaccines in C57BL/6 mice did not improve FA. Equally, similar FA was observed after vaccination with virus‐like particles displaying low versus high antigen densities. Interestingly, FA was stable in vivo but not in vitro, depending on the antigen dose and the time interval since T‐cell activation, as observed in murine monoclonal T cells. Our findings suggest dynamic in vivo modulation for equal FA. We conclude that low antigen density vaccines or a minimal 4‐week prime/boost interval are not crucial for the T‐cell's FA, in contrast to antibody responses.

## Graphical abstract

Vaccination with changes in the prime/boost interval, or antigen density, did not influence the functional avidity (FA) of antigen‐specific CD8 T cells. However, a monoclonal population in vitro showed improved FA over time. These results suggest steady FA regulation in vivo to ensure equal and stable function and efficiency.

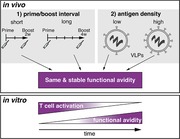

## Introduction

It has long been known that B‐cell responses employ affinity maturation to optimize antibody affinity over time 1, 2. Consequently, most vaccine regimens are designed to induce B‐cell responses with favorable antibody affinity, by using vaccines with well‐chosen antigen doses/densities, and by vaccination with prime/boost (P/B) intervals of at least 4 weeks. The situation for improving the quality of T cells is much less clear 3. The functional avidity (FA) describes the T‐cell's sensitivity for peptide‐MHC (pMHC) and correlates with biological functions such as killing, cytokine production, and anti‐viral protection 4. Several studies showed that during a CD8 T‐cell response, the clonality and the FA remains remarkably stable 5, 6, 7, 8, 9. Other studies demonstrated FA changes over time, in part due to shifts in the clonal dominance and thus in the TCR usage 10, 11.

Even though TCR affinity and T cells’ FA are important correlates of protection from disease 4, 12, it remains poorly understood whether certain vaccine doses, formulations, or P/B schemes induce T‐cell responses with better affinity and FA. Some researchers have primed with one vaccine, only to boost with a different vaccine formulation optimized for CD8 T‐cell responses with enhanced FA 13. Many studies investigating changes in the FA of the peptide‐specific CD8 T‐cell response have focused on altered peptide ligands to artificially modulate the TCR pool recruited. Although the altered peptides were primarily designed for improved peptide‐MHC binding affinity, they often displayed small but relevant alterations in TCR binding 14, 15. However, changing epitopes from prime to boost and/or effector phase preclude direct conclusions on eventual avidity maturation because avidity differences may be due to fine specificity differences.

The question as to whether avidity maturation occurs in vivo, and if so can be improved by optimizing vaccination dosing and scheduling, can be better addressed by using the same vaccine formulation for sequential “homologous” vaccinations, focusing on precisely the same epitope. There has been evidence from early studies suggesting that using low antigen doses produced T cells with higher avidity, compared to high dose 16, 17, 18, 19. However, others have shown that it was the dose used for a boosting immunization that was critical to achieve improved FA, with no difference after a single immunization 20 and a more recent study found that CD8 T‐cell FA was not altered by changes in vaccine dose 21. Therefore, it remains unclear exactly how changes in peptide dose influences the FA of a peptide‐specific T‐cell response to vaccination. Two studies investigating T‐cell responses to infection with *Listeria monocytogenes* and LCMV, respectively, showed evidence for avidity maturation 22, 23. The latter study suggested FA maturation during the first week of priming in a monoclonal CD8 T‐cell population 23. However, the practical question remains open whether short‐term homologous P/B vaccinations with subunit vaccines can be optimized to achieve high FA T‐cell responses, through strategies analogous to vaccination for high affinity antibody responses. Therefore, we used subunit vaccines to investigate whether homologous vaccination with different P/B intervals (2 vs. 4 weeks) or altered antigen density would impact the FA of a peptide‐specific CD8 T‐cell response.

## Results

### Functional avidity (FA) was not improved with a prolonged prime/boost (P/B) interval

**Figure 1:**
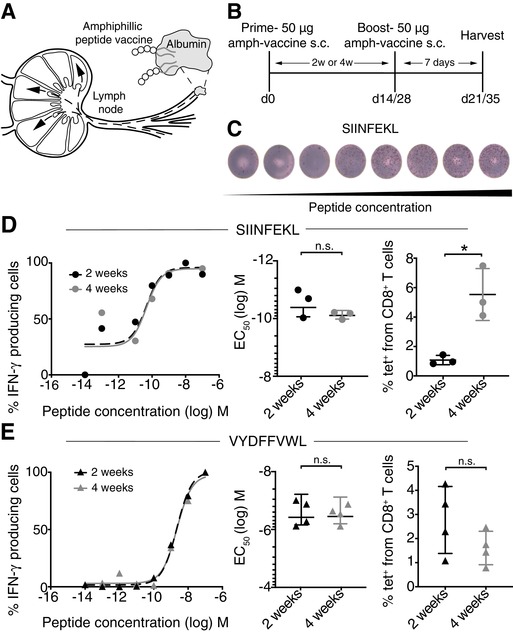
Delayed boost vaccination does not improve functional avidity (FA) of ovalbumin‐specific CD8 T cells. (A) Schematic of amph‐vaccine design. (B) WT mice were immunized s.c. at the tail base with 50 µg amph‐vaccine containing SIINFEKL (ovalbumin) peptide and boosted with the same dose at either 2‐ or 4‐week following the prime. Splenocytes were harvested 7 days following last vaccination. (C) Representative wells from one titration (performed in triplicates) of an IFN‐γ ELISpot assay. (D) A dose titration of SIINFEKL peptide was used to determine FA, that is the peptide dose (EC_50_) required for half maximal IFN‐γ ELISpot forming cells. The data are from a single experiment representative of two independent experiments (*n* = 3 mice/experiment). Blood was taken 7 days following the boost and analyzed by flow cytometry. Cells were gated on K^b^‐SIINFEKL tetramer positive cells from CD8^+^CD3^+^ T cells, as shown in Supporting Information Figure 1. Statistical analysis by unpaired *t*‐test, *p < 0.05. (E) Mice were immunized s.c. at the tail base with 50 µg of amph‐vaccine containing VYDFFVWL (Trp2) peptide, and the ELISpot titrated with VYDFFVWL peptide. Blood was taken 7 days following the boost and analyzed by flow cytometry. Cells were gated on K^b^‐VYDFFVWL tetramer positive cells from CD8^+^CD3^+^ T cells. The data are combined from two independent experiments (*n* = 2 mice/experiment). Statistical analysis by unpaired *t*‐test. All values show mean and SD.

Based on the knowledge of vaccination for antibody responses, we wanted to investigate the effect of boosting at different time points after an initial prime. It was of interest to determine whether the FA of CD8 T cells would be improved after a 4‐week delay compared to 2‐week, as a minimal 4‐week interval is standard clinical practice for vaccinations inducing antibody responses (https://www.cdc.gov/vaccines/hcp/acip-recs/index.html). To address this question, WT mice were primed s.c. with 20 µg of the potent subunit amphiphilic vaccine (Amph‐vaccine) containing the ovalbumin epitope, SIINFEKL, and cyclic di‐GMP, as an adjuvant for LN targeting (Fig. 1A) 24, 25. A boost vaccination of the same dose was given either 2 or 4 weeks following the prime (Fig. 1B). Splenocytes were harvested 7 days following the boost and directly plated in an IFN‐γ ELISpot assay with soluble peptide (Fig. 1C) to determine the peptide dose for the half maximal response (EC_50_), reflecting the FA. When comparing mice that received either a 2‐ or 4‐week boost, there was no difference in the mean EC_50_ (Fig. 1D). However, a 4‐week boost improved the quantity of peptide‐specific CD8 T cells compared to the 2‐week boost (Fig. 1E, Supporting Information Fig. 1A). Despite the increased number of tetramer positive CD8 T cells, FA was not improved. The P/B regimen was also tested using an amph‐vaccine with the tyrosine‐related peptide 2 (Trp2) (VYDFFVWL). Similarly, we found no difference in EC_50_ between a 2‐ or 4‐week boost (Fig. 1F). Here, we found similar numbers of tetramer positive CD8 T cells (Fig. 1G, Supporting Information Fig. 1B). With these two amph‐vaccines, the mean EC_50_ of the IFN‐γ response was comparable between a 2‐ or 4‐week boost. Thus, in contrast to what is known for B‐cell response‐inducing vaccines, these results indicate that delaying the second vaccination from 2 to 4 weeks does not improve the FA of the peptide‐specific T‐cell response.

### Vaccinations with virus‐like particles (VLPs) coated with different peptide densities do not affect the FA

**Figure 2:**
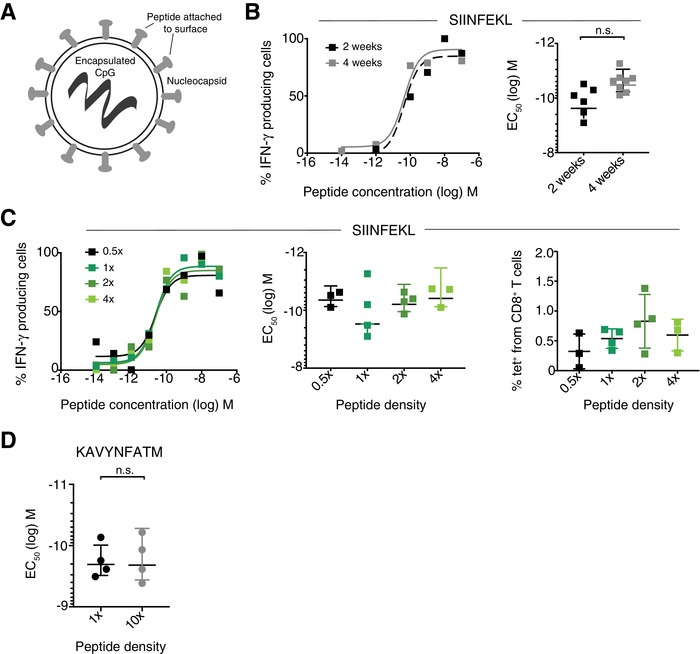
Similar FA induced by vaccinations with VLPs despite different prime/boost (P/B) interval or antigen density. (A) Schematic of VLP vaccine design. (B) WT mice were immunized s.c. at the tail base with 50 µg VLP peptide displaying SIINFEKL and boosted with the same dose at either 2‐ or 4‐week following the prime. Splenocytes were harvested 7 days following last vaccination and analyzed in an IFN‐γ ELISpot assay with titrated SIINFEKL peptide. Statistical analysis by unpaired *t*‐test. The data are combined from two independent experiments with different VLP densities used (*n* = 1–2 mice per density/experiment). (C) Vaccination with VLPs displaying SIINFEKL peptide at peptide densities of 0.5, 1, 2, or 4×, respectively. Schedule as above, with a boost at 2 weeks. Statistical analysis by one‐way ANOVA with Bonferroni post‐test. All comparisons were not significant. Blood was taken 7 days following the boost and analyzed by flow cytometry. Cells were gated on K^b^‐SIINFEKL tetramer positive cells from CD8^+^CD3^+^ T cells, as shown in Supporting Information Figure 1. The data are combined from two independent experiments (*n* = 2 mice per density/experiment). (D) Vaccination with VLPs displaying KAVYNFATM (LCMV gp33) peptide. Schedule as above. Analysis with an ELISpot assay with titrated KAVYNFATM peptide. The data are combined from two independent experiments (*n* = 2 mice/experiment). Statistical analysis by unpaired *t*‐test. All values show mean and SD. *p < 0.05.

A third subunit vaccine was used, composed of virus‐like particles (VLPs) containing type B CpG ODN as an adjuvant and SIINFEKL peptide (Fig. 2A) 26. We first compared the P/B regimen and again found no difference in EC_50_ between a 2‐ or 4‐week boost (Fig. 2B). Using VLPs allowed us to investigate changing the antigen density, as previous research has suggested that increased antigen availability lowers the FA of the corresponding CD8 T cells 16, 27. Using VLPs with a range of SIINFEKL peptide densities (0.5–4‐fold, Supporting Information Fig. 2), mice were immunized s.c., boosted at 2 weeks with the same density as the prime, and spleens were harvested 7 days following the boost for FA assessment by IFN‐γ ELISpot assay. There was no difference in EC_50_ when comparing the different antigen densities (Fig. 2C). The magnitude of the peptide‐specific T‐cell response between the different VLP densities was also unchanged (Fig. 2C, Supporting Information Fig. 1C). Therefore, the FA of the CD8 T‐cell response was not altered by vaccinations with different peptide densities. Indeed, when testing the same regimen with mice immunized with VLPs loaded with the gp33 peptide (KAVYNFATM) and a 10× difference between peptide densities, the FA remained the same (Fig. 2D). These results indicate that responding CD8 T cells have similar FA despite the P/B vaccinations with different peptide densities.

### FA improves in vitro with increased time after activation

**Figure 3:**
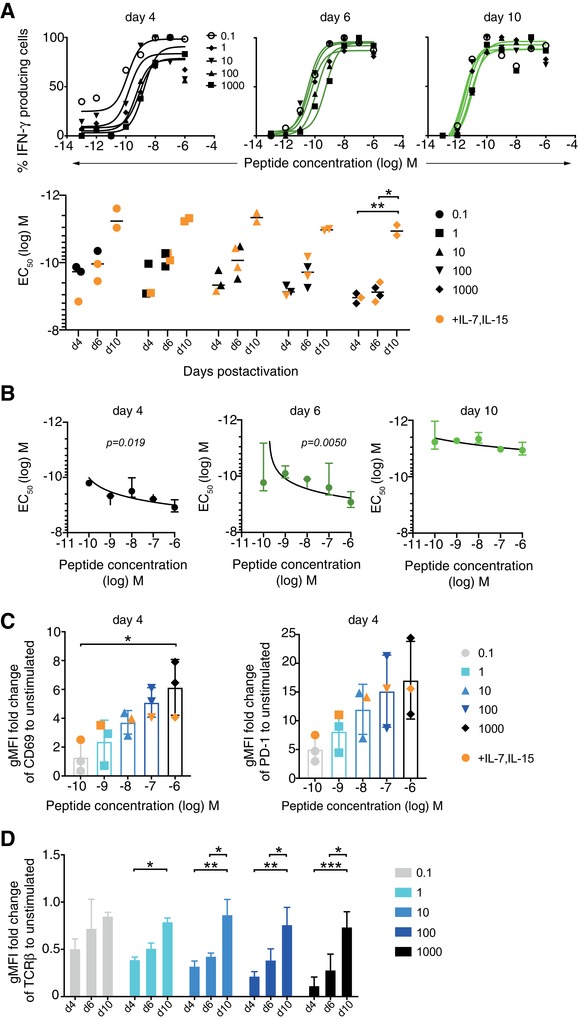
FA increases in vitro with time after peptide activation. (A) Splenocytes from untreated OT‐1 mice were cultured with a range of SIINFEKL peptide doses (0.1 to 1000 nM). Cells were collected at days 4, 6, and 10 postactivation for IFN‐γ ELISpot assay with titrated peptide concentrations (*x*‐axis) and parallel flow cytometry analysis. Cells were cultured with hIL‐2 alone, or hIL‐2 with hIL‐7 and hIL‐15 as depicted in orange. The data are combined from three independent experiments with triplicates/experiment, with representative titration curves from one. Statistical analysis by two‐way ANOVA with Bonferroni post‐test. (B) Correlations of FA with the peptide concentration used for in vitro activation of OT‐1 splenocytes. (C‐D) Cell surface expression of CD69, PD‐1, and TCRβ determined by flow cytometry and presented as geometric MFI (gMFI) fold change compared to unstimulated control cells. The data are combined from two to four independent experiments with one sample/experiment. Statistical analysis by two‐way ANOVA with Bonferroni post‐test comparing time. All values show mean and SD. *p < 0.05, **p < 0.01, ***p < 0.001.

The second half of our study focused on monoclonal T cells, to investigate TCR affinity‐independent FA regulation in response to homologous peptide vaccination. It has previously been shown that a monoclonal T‐cell population was able to undertake avidity maturation during the first few days after LCMV infection, based on TCR affinity‐independent FA regulation 23. We wondered if there was an early modulation of the T‐ cell response that eventually “leveled out” to a moderate FA that remained stable. To determine whether this may occur with a monoclonal T‐cell population, we set up in vitro cultures using monoclonal OT‐1 T cells specific for SIINFEKL presented on H2‐K^b^. OT‐1 splenocytes were activated with a range of SIINFEKL peptide concentrations (0.1 to 1000 nM) and cultured with either IL‐2 alone, or IL‐2 with IL‐7 and IL‐15 to improve viability, which was required for longer cultures (day 10). Cells were harvested at days 4, 6, and 10 postactivation to assess FA by IFN‐γ ELISpot assay, and cell surface receptor expression was evaluated concurrently by flow cytometry analysis. We found that the EC_50_ improved with time by over one log, an effect that was statistically significant when the cells had been activated with 1 µM peptide, and with the same trend for all other peptide doses tested (Fig. 3A). The addition of IL‐7 and IL‐15 for cell survival did not impact the FA observed. Most of the FA improvement occurred between day 6 and day 10. These results are comparable to our previous findings showing that the FA of human T‐cell clones was related to the cellular activation status in vitro. Supporting Information Fig. 3 shows these previously published data 28 in more detail, where peptide‐specific CD8 T‐cell clones were cultured from the PBMC of vaccinated melanoma patients. These results emphasize that human CD8 T‐cell clones consistently increase their FA from day 10 to day 15 after restimulation, in parallel to the steady reduction of their activation state 28.

Besides the influence of time postactivation on FA, we also found a minor but consistent effect of the peptide dose used to activate OT‐1, with decreasing EC_50_ as peptide dose increases (Fig. 3A and B). As expected, we observed regulation of coreceptors with rapid activation, as seen by the increased expression of CD69 relative to unstimulated cells, as well as a trend for increased PD‐1, in a dose‐dependent manner (Fig. 3C, Supporting Information Fig. 4). Previous studies have shown that the strength of stimulus will cause a concentration dependent downregulation of TCRβ 29, which is known to directly impact the FA 30. As anticipated, we saw that higher peptide concentrations induced stronger TCRβ downregulation, followed by TCRβ increase over time (Fig. 3D).

### FA remains stable in vivo

**Figure 4:**
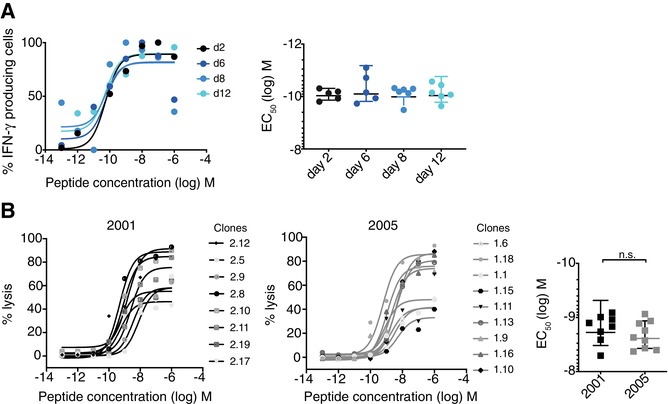
FA remains stable in vivo. (A) Purified CD8^+^ OT‐1 cells (1 × 10^6^) were adoptively transferred into WT mice. Six hours following transfer, mice were vaccinated s.c. at the tail base with 10 µg SIINFEKL and 50 µg CpG 7909. Splenocytes were harvested at various time points and analyzed in an IFN‐γ ELISpot assay. Left: Representative titration curves. Right: The data are combined from two independent experiments (*n* = 2–3 mice/experiment, mean values ± SD). Statistical analysis by one‐way ANOVA with Bonferroni post‐test. There were no statistically significant differences. (B) Human HLA‐A2/NY‐ESO‐I‐specific CD8 T‐cell clones were generated using PBMC obtained in the years 2001 and 2005 from a melanoma patient. The clones were tested in cytotoxicity assays using T2 target cells pulsed with titrated NY‐ESO‐1 peptide to determine the FA (EC_50_). The data are combined from three to four independent experiments with triplicates/experiment (*n* = 8–10 clones/experiment, mean values ± SD). Statistical analysis by Mann–Whitney test.

We then wanted to know whether these early FA changes of a monoclonal population observed in vitro were similarly found in vivo early after vaccination. Purified CD8 OT‐1 cells were adoptively transferred into WT mice that were immunized with CpG and SIINFEKL (the same peptide as used in vitro) 6 h following transfer. Cells were harvested at various days postactivation to assess FA by IFN‐γ ELISpot assay. Interestingly, when comparing the earlier (days 2 and 6) and the later days (days 8 and 12) postimmunization, the EC_50_ remained unchanged (Fig. 4A).

For many years, we have been characterizing the FA of CD8 T cells in healthy volunteers and cancer patients. We and others have shown that in chronic antigen exposure, long‐term antigen‐specific CD8 T cells also demonstrate remarkable FA stability and clonotypic stability 5, 6, 7, 8, 9. In addition, here we studied a melanoma patient who had developed a strong immune response by expansion of HLA‐A2/NY‐ESO‐1‐specific CD8 T cells, as described previously 31. T‐cell clones were generated from PBMC taken at two different collection times, and their FA was determined. Interestingly, we found similar EC_50_ values with the clones generated from PBMC obtained in the year 2001, as compared to those of the year 2005 (Fig. 4B). The repertoire at the two time points showed sharing of several clonotypes and TCR β‐chain subfamilies (Supporting Information Table 1). There was no evidence of enrichment of higher affinity T cells. This stability is in line with the majority of findings in the literature, showing that viral antigen‐specific CD8 T‐cell responses in vivo have remarkably stable FA 8, 9.

In summary, our in vitro data showed increased FA with time after activation and confirmed findings in the literature showing that FA is modulated by TCR affinity‐independent regulators 23, 32, 33. However in vivo, we observed FA stability despite different P/B intervals and peptide densities, and found FA stability over time; suggesting that FA regulators are constantly active in vivo to assure stable and equal FA.

## Discussion

Using subunit vaccines, we addressed the impact of homologous P/B regimens and different antigen density on the FA of peptide‐specific CD8 T cells. Despite its practical importance, it has remained unclear whether the length of the P/B interval is critical for optimal FA for a T‐cell response to a given peptide antigen 34. In clinical practice, a short interval would be easier, as compared to the minimal 4‐week period that has been widely accepted for inducing high affinity antibody responses. Whether this would be applicable to the T‐cell response has yet to be determined, specifically with homologous vaccination. To investigate, we assessed both 2‐ and 4‐week boosting after prime, with the prediction that a 2‐week boost would be suboptimal. Interestingly, we found no difference in the FA of the peptide‐specific CD8 T‐cell response, indicating that the 4‐week interval does not mediate a more favorable FA.

The FA depends on the TCR affinity to the pMHC. In murine and human CD8 T‐cell responses, some studies showed increasing dominance of cells bearing TCRs mediating stronger T‐cell functions 10, 35, 36. Thus, changes in clonal dominance may impact on the FA. While most studies relied on partial characterization of the involved TCRs 22, full characterization of TCR‐α and ‐β chains and the role of CD8 binding showed clonal shifts in some studies 37, 38, in contrast to stability in others 5, 6, 7, 8. Shifts of codominant clones usually occur over longer periods of time, typically between the initial T‐cell response and the memory response many weeks later 10, 39, 40. Our study is not focused on memory (long‐term) responses, but on the generation of the primary immune response. In this short time‐frame, one would not expect clonotypes (with TCRs mediating similar function) to show early short‐term selection. Even after long intervals, the TCR repertoire and consequently the FA can remain highly stable as found in some models or patients 5, 7, 37, whereas long‐term selective outgrowth of clonotypes with different TCR affinity and different FA may occur in other settings 9, 41.

It has been suggested that the use of low antigen dose may achieve high FA responses 16, 19. A frequently cited study with DCs loaded with high versus low peptide concentrations showed more favorable FA induced by low peptide 17. However, another study found that DCs loaded with higher antigen did not impact on FA, whereas it improved T‐cell expansion 42. In the context of peptide vaccination, one study found that low antigen dose improved the FA of CD4 T cells, but this did not extend to CD8 T cells 21. To vary antigen dose in vivo, we designed a novel approach, consisting of VLPs decorated with different peptide densities. The presumption made is that high peptide density gives rise to higher peptide presentation by APCs in vivo. Due to the technical challenges involved, the peptide density on the surface of DCs is rarely determined including the study cited above 17. Moreover, DC vaccination is not broadly applicable and using low peptide doses makes it difficult to verify peptide density in vivo. We used VLPs with different peptide density within a 10‐fold range to reflect what can practically be achieved in vaccines with potential clinical application. Interestingly, the FA was not improved by vaccination with low peptide density VLPs.

It is worth noting that the stable FA observed is within the context of homologous subunit vaccination; different results may be obtained with other types of immunization, and/or when using different vaccines for P/B. Using heterologous P/B vaccination, several studies have shown that different vaccine formulations and epitopes used for boosting can achieve improved FA 13, 14, 15, 43. However, boosting with a vaccine that differs from the prime biases the T‐cell pool and may select for those with higher affinity, reflecting the differences of the vaccines used rather than endogenous avidity maturation alone. Furthermore, our study did not aim at optimizing the magnitude of the CD8 T‐cell response as it does not necessarily equal protective capacity, at least not when comparing immunization strategies that all reach high frequencies 44. While we focused on the FA in response to subunit vaccination, other studies analyzed the FA in response to infections. Slifka *et al*. has shown FA maturation in a T‐cell response to LCMV infection 23, and other studies used *L. monocytogenes* and influenza infections to demonstrate T‐cell affinity maturation through clonal shifts 10, 22. The increased inflammation that occurs during infection may impact on the FA, as it has been shown that effector functions were enhanced in both low and high affinity T cells in mice that were administered peptide‐pulsed DCs with a concurrent *L. monocytogenes* infection, compared to no infection 45.

A central finding of our study is that the FA of OT‐1 cells underwent considerable changes in vitro, as opposed to the observed FA stability in vivo. Since these monoclonal T cells are controlled through a single type of TCR, the FA differences observed in vitro are TCR affinity independent. FA changes in vitro depended on the timing postactivation, and the antigen concentration used, which both impact the activation‐dependent changes in receptor levels involved in the immune synapse, and consequently the FA. Similar activation state‐dependent FA changes can also be observed in human CD8 T‐cell clones analyzed in vitro 28. These results also point to an important methodological aspect: in order to obtain reproducible FA data, T‐cell clones must always be analyzed after the same number of days since their last restimulation. The recent introduction of *K*_off_‐rate measurement using new generation fluorescent tetramers 28, 46 represents clear progress in the development of new methods to assess T‐cell avidity. Importantly, *K*_off_‐rate data is independent of activation and also independent of synapse molecules and coreceptors (besides CD8). While these techniques are excellent for assessing monoclonal T cells, they are unfortunately not yet satisfactory for the characterization of the complex natural polyclonal T‐cell responses.

We think that the remarkable FA stability observed in vivo after early peptide vaccination may involve constant synapse regulation. Synapse alterations are more flexible and, thus, more prominent as opposed to TCR affinity changes (i.e. clonal shifts), particularly for rapid avidity regulation, which is likely involved for keeping FA stable in vivo. There are multiple receptor‐ligand pairs in the immune synapse that may impact the FA 33, 47, 48 such as regulatory receptors like PD‐1. One study used a poxvirus to infect APCs to express a triad of ICAM‐1, LFA‐3 and CD80, which led to enhanced high avidity CD8 T cells in vivo, both in number and function. This improvement was observed in both primary and secondary responses 49. Many excellent studies have characterized the various components of the complex synapse mechanisms 49, 50, 51. Nevertheless, it is probably still a long way until one can quantify the roles and contributions of each component and, thus, fully understand how FA is regulated by the synapse.

The potential flexibility of synaptic components to alter FA in vivo may have the aim to give a close‐to‐equal chance to T cells with different affinity TCRs, at least for those within a relatively narrow affinity range mediating equal function 52. A powerful T‐cell response usually involves broad polyclonal involvement, to have many T cells participating in an epitope‐specific response. Indeed, polyclonality has been identified as a correlate of protection 53, 54. It seems possible that FA regulation and stability is required to avoid major selective outgrowth of very few clones at the expense of most others. Modulation for equal FA in vivo may also apply to T cells with the same TCR, to compensate the consequences of their varying differentiation and activation states (e.g. by different degrees of TCR downregulation) that have the potential to alter their functionality. Conceivably, modulation occurs in such a way that T cells have equal functional interactions with cells presenting cognate antigen, irrespective of their levels of activation and differentiation.

We conclude that subunit vaccination induces CD8 T‐cell responses with in vivo stable FA over time, in contrast to affinity maturation observed in antibody responses. This suggests that it is inconsequential to choose 2‐ or 4‐week intervals, or a particular antigen density on vaccine particles. For clinicians, this may be welcome news as it implies that 2‐week boosting is acceptable, thereby, greatly reducing the downtime required between immunizations, which often must be repeated multiple times for sustained T‐cell responses. Our results advocate for a T‐cell population capable of adapting FA to ensure equal efficiency in vivo.

## Materials and methods

### Mice

C57BL/6JOlaHsd mice were purchased from Envigo (Bicester, UK). OT‐1 mice were originally from The Jackson Laboratory and bred in‐house. All animal experiments were approved by the Veterinary Authority of the Swiss canton Vaud and performed in accordance with Swiss ethical guidelines. Mice were age matched, female and at least 6 weeks old at the beginning of the experiment, and maintained in conventional facilities at the Epalinges site of the University of Lausanne (UNIL).

### Vaccines

Mice were treated with 10 µg of SIINFEKL peptide (in‐house peptide facility) with 50 µg of CpG oligodeoxynucleotide 7909 (CpG ODN). Treatments were injected at the tail base s.c. in a total volume of 100 µL. Amphiphilic vaccines contained 20 µg of SIINFEKL or 20 µg of VYDFFVWL, and 25 µg of cyclic di‐GMP as an adjuvant (Amph‐vaccine). Virus‐like particles (VLPs) were produced with the bacteriophage Qβ protein, and packaged with the type B CpG ODN 1668 (1.125 µg per 20 µg Qβ), administered at a dose of 50 µg/mouse. The VLPs were coupled with different excess of the linker SMPH to achieve different surface peptide densities. A 4× excess of SIINFEKLGGC‐OH peptide (Pepscan) was then bound to the SMPH, or 4× excess of KAVYNFATMGGC‐OH (LCMV gp33) peptide (Pepscan, Lelystad, The Netherlands).

### ELISpot assay

FA was always assessed 7 days after the last boost to compare immunizations. Splenocytes were plated in a range of concentrations (2 × 10^3^ to 1 × 10^5^/well) on Multiscreen HTS plates (Merck, NJ, USA) using the mouse IFN‐γ ELISpot BASIC (ALP) kit to detect murine IFN‐γ (Mabtech, Stockholm, Sweden). Cells were stimulated with titrated soluble peptide. As a positive control, cells were stimulated with PMA 50 ng/mL and Ionomycin 500 ng/mL (both Sigma‐Aldrich, St. Louis, MI). Background from control wells (unstimulated cells) were subtracted from the spot forming units counts. Counts were normalized to graph as a percentage of IFN‐γ producing cells and nonlinear regression curves were used to calculate EC_50._

### Adoptive transfer of OT‐1 cells

CD8 T cells were purified from naive OT‐1 splenocytes using the EasySep™ Mouse CD8 T‐cell Isolation kit (StemCell, Vancouver, Canada). A total of 1 × 10^6^ purified cells were transferred into WT mice and injected i.v.

### In vitro stimulation of OT‐1 cells

Splenocytes were stimulated with various doses of soluble SIINFEKL peptide (0.1 to 1000 nM) and incubated at 37°C in 10% FCS, 5 mg/mL penicillin, 5 mg/mL streptomycin, 0.05 mM 2‐ME, 10 mM HEPES, and 1 mM sodium pyruvate (all Invitrogen, CA, USA) in RPMI (cRPMI). Cells were split on day 4, following peptide stimulation and 20 U/mL of hIL‐2 (Roche) was added to each well. Long‐term cultures were split every other day, starting from day 2, into cRPMI with 10 U/mL of hIL‐2, 10 ng/mL of hIL‐7 (Miltenyi Biotec, Bergisch Gladbach, Germany) and 10 ng/mL of hIL‐15 (Peprotech, Rehovot, Israel). Unstimulated cells were cultured in the same conditions without peptide and were used as controls.

### Flow cytometry

Single cell suspensions were resuspended in FACS buffer (PBS with 10 mM EDTA, 2% FBS) before staining with the following antibodies: CD3 (17A2) that was prepared in‐house, PD‐1 (29F.11A12), CD8 (53–6.7) from Biolegend and TCRβ (H57.597), CD69 (H1.2F3) from eBioscience. Fresh blood from mice was lysed with red blood cell lysis buffer (in‐house) and washed twice with FACS buffer before staining as above. Peptide/H2‐K^b^ tetramers were made in‐house. Dead cells were excluded as staining positive with LIVE/DEAD™ Fixable Aqua Dead (Thermo Fisher Scientific, Waltham, MA). Cells were washed and resuspended in FACS buffer. Acquisition was performed on a BD LSRII SORP (Becton Dickinson, NJ, USA) or a BD LSR Fortessa SORP (Becton Dickinson) and data were analyzed using FlowJo version 10.3 (Tree Star). All experiments were performed according to guidelines for the use of flow cytometry and cell sorting in immunological studies 55.

### Melanoma patients and human CD8 T cells

Melanoma patients provided written informed consent and participated in clinical investigation protocols of the Ludwig Institute for Cancer Research (LICR), approved by the Institutional Review Boards and the LICR Protocol Review Committee. Blood samples were collected and processed as previously described 31. CD8 T cells were sorted and cloned by limiting dilution and expanded, and the FA was determined by peptide titration killing assays as previously detailed 31.

### Statistics

Statistical analyses were performed with GraphPad Prism 8. Number of spot forming units were normalized and EC_50_ values were calculated using nonlinear regression curves. Normality was assessed using the Shapiro–Wilk test, with data having passed the normality check. Therefore, statistical evaluation was by unpaired *t*‐test, one‐way or two‐way ANOVA with the Bonferroni correction. Patient data were analyzed by the Mann–Whitney test. Differences of *p *< 0.05 were deemed statistically significant.

## Conflict of interest

The authors declare no commercial or financial conflict of interest.

**Abbreviations**
ALPs: altered peptide ligands
FA: functional avidity
LCMV: lymphocytic choriomeningitis virus
P/B: prime/boost
pMHC: peptide‐MHC
VLPs: virus‐like particles

## Supporting information

Supporting Information
