# Supplementary material for: Murine CD8 T‐cell functional avidity is stable in vivo but not in vitro: Independence from homologous prime/boost time interval and antigen density
Source: Eur J Immunol. 2019 Dec 10;50(4):505–14. doi: 10.1002/eji.201948355 (PMC7187562; doi:10.1002/eji.201948355)
Supplement: Supplementary file 1 — Supporting Information [file EJI-50-505-s001.pdf]

Supporting Information

Gilfillan et al.,

Supporting Information Figure 1

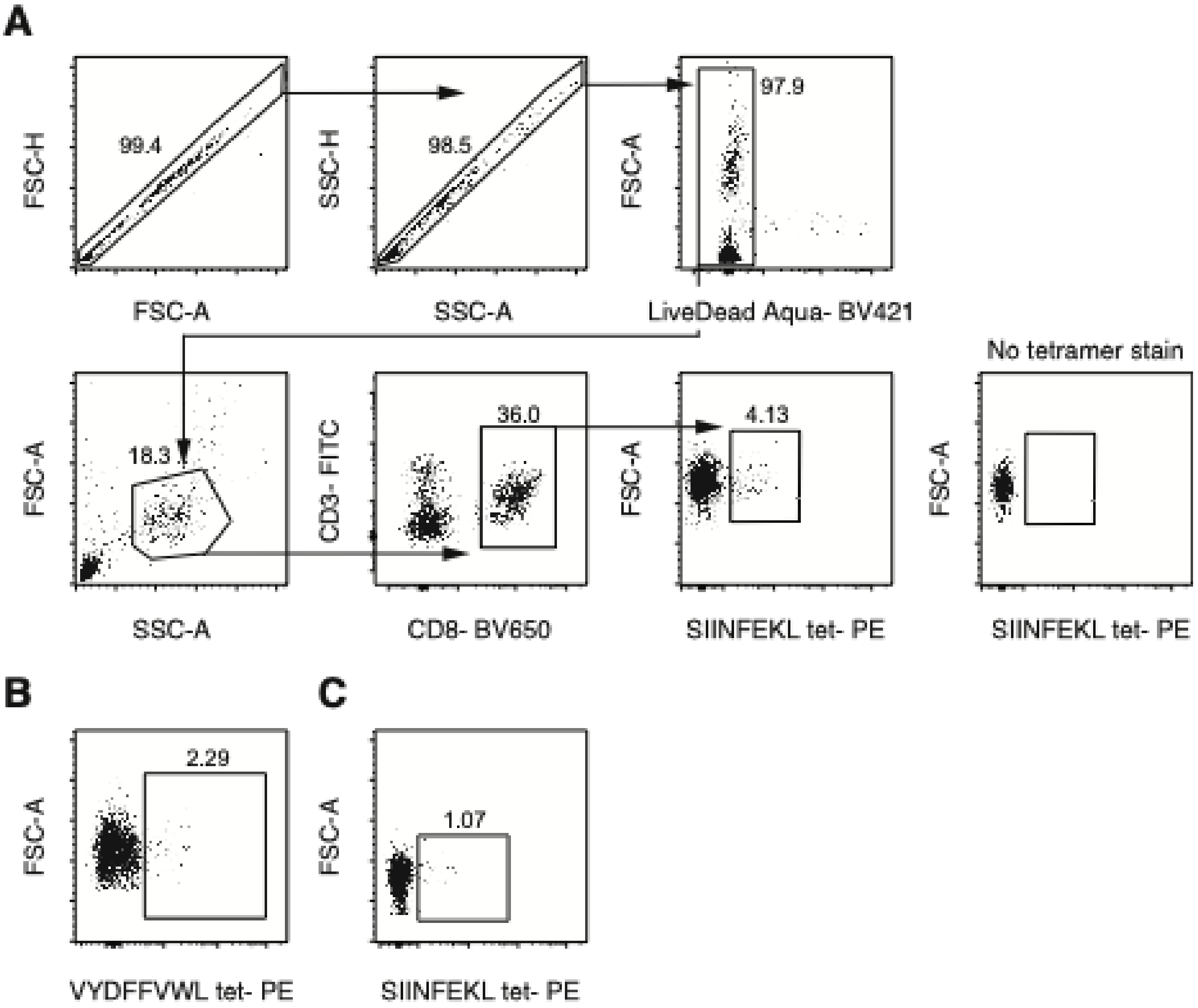

Supporting Information Figure 1: Gating strategy for FACS analysis of tetramer positive CD8 T cells in vaccinated mice. A) Blood samples taken from vaccinated mice were processed as described in Material and Methods. Cells were gated on singlets, live, lymphocytes and $CD8^+CD3^+$, successively. SIINFEKL-H2-K_b tetramer conjugated to PE was used to identify peptide-specific CD8 T cells from mice vaccinated with amph-vaccine containing SIINFEKL. Cells unstained with tetramer were used as controls for gating for all experiments. B) VYDFFVWL-H2-K_b tetramer was used for mice vaccinated with VYDFFVWL containing amph-vaccine. C) SIINFEKL-H2-K_b tetramer was used for mice vaccinated with VLPs containing SIINFEKL.

Supporting Information Figure 2

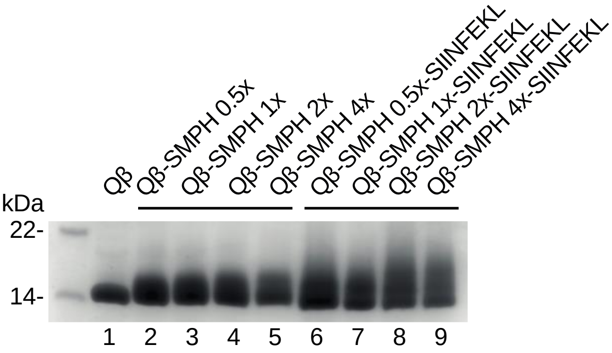

Supporting Information Figure 2: Coupling of SIINFEKL peptide using SMPH cross-linker. SDS-PAGE of Qβ-VLPs. 1) Qβ monomer, MW of 14kDa. 2-5) Qβ coupled to SMPH linker at densities of 0.5x-4x. 6-9) Qβ-SMPH 0.5x-4x, each coupled with 4x excess SIINFEKL peptide. Unbound linker is eluted before coupling to peptide, hence linker density reflects peptide density.

Supporting Information Figure 3

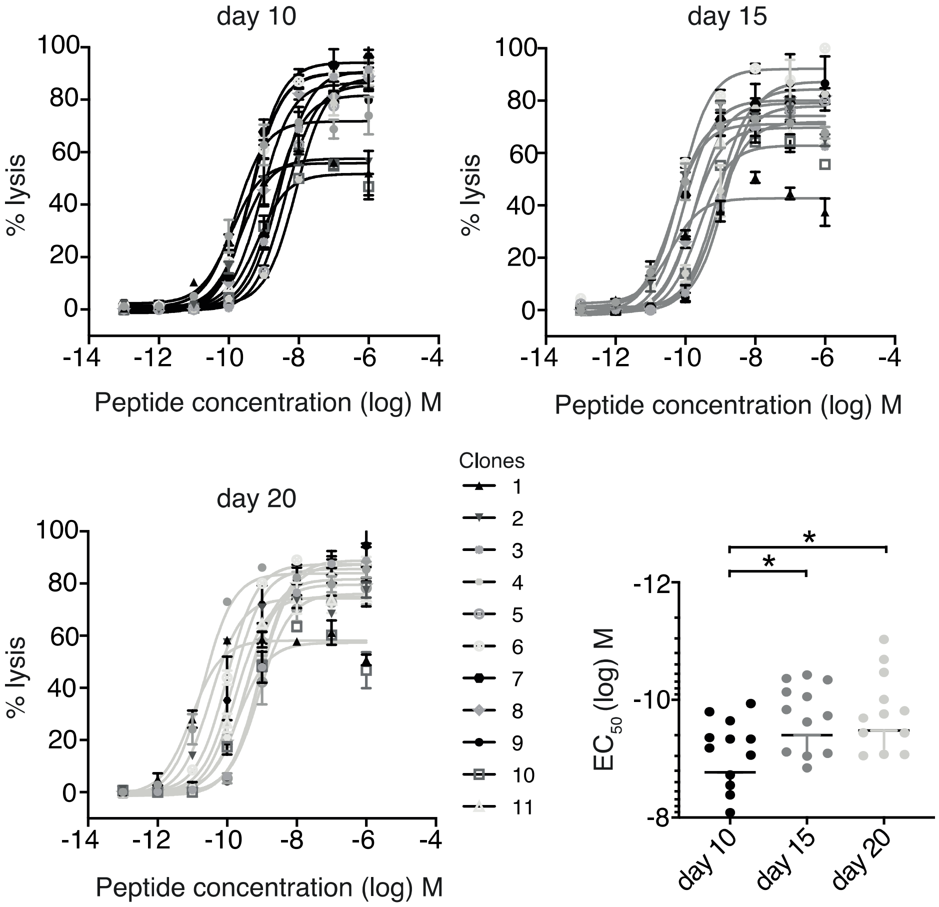

Supporting Information Figure 3: Improved FA of 11 T cell clones post in vitro activation. CD8 T cell clones specific for HLA-A2/Melan-A were generated from PBMC of melanoma patients vaccinated with Melan-A peptides as described (1). For maintenance, the clones were restimulated with PHA and irradiated feeder cells every 3 weeks. With this method, the clones can be used for functional assays between 10 and 20 days after restimulation, i.e. in the time window when enough cells are available in good quality. Results are shown of cytotoxicity assays against T2 target cells pulsed with titrated concentrations of Melan-A peptide (29). The assay was done 10, 15 and 20 days after restimulation of the T cell clones, showing progressive FA increase, primarily between days 10 and 15. Statistical analysis by two-way ANOVA with Turkey post-test. All values show mean and SD. *p <0.05.

Supporting Information Figure 4

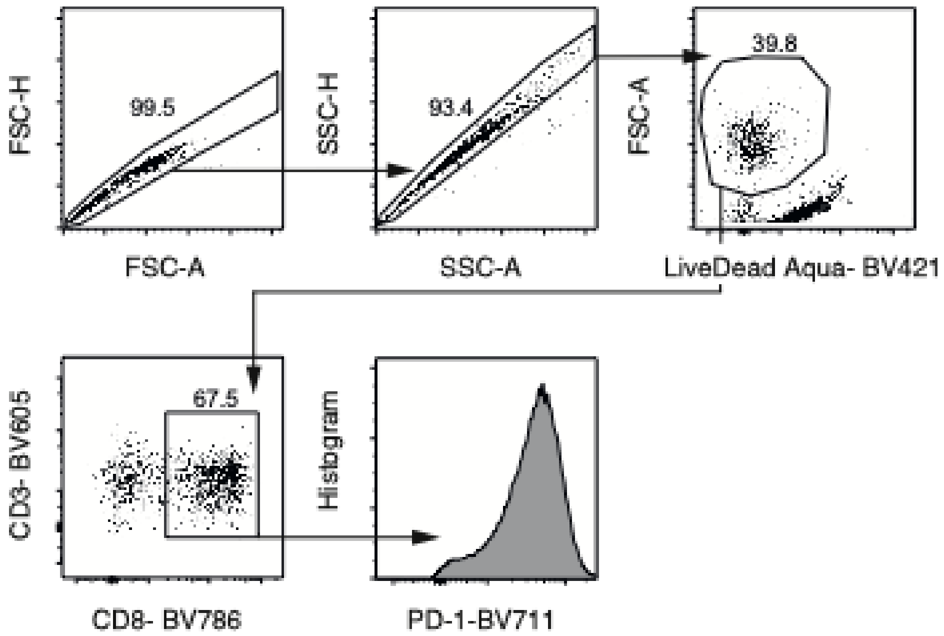

Supporting Information Figure 4: Gating strategy for FACS analysis of cultured OT-I cells. Splenocytes from OT-I mice were cultured with a range of SIINFEKL peptide doses, IL-2 and with or without IL-7 and IL15. Cells were collected at days 4, 6 and 10 post-activation for FACS analysis. Cells were gated on singlets, live and $CD8^{+}CD3^{+}$, successively. Shown above is a representative gating of CD8 cells (day 6, 1 μM stimulation, IL-2). The gMFI was taken for various receptors (PD-1, CD69, TCRβ) as described and shown in Fig. 3.

1. Allard M, Couturaud B, Carretero-Iglesia L, Duong MN, Schmidt J, Monnot GC, Romero P, Speiser DE, Hebeisen M, Rufer N. 2017. TCR-ligand dissociation rate is a robust and stable biomarker of CD8+ T cell potency. *JCI Insight*. 2.

Supporting Information Table 1

β-chains of the NY-ESO-1 specific CD8 T cell clones

Year	BV (Clone name)	Year	BV (Clone name)
2001	BV1-2 (2.12)	2005	BV1-2 (1.6)
	BV8-1 (2.5)		BV1-2 (1.18)
	BV8-2 (2.9)		BV8-1 (1.11)
	BV8-3 (2.8)		BV8-1 (1.13)
	BV8-3 (2.10)		BV2 (1.1)
	BV8-3 (2.11)		BV2 (1.15)
	BV8 other (2.19)		BV13-1 (1.9)
	BV13-2 (2.17)		BV13-1 (1.16)
			BVx (1.10)

Details of the CD8 T cell clones used in the experiment shown in Fig. 4B. Clones with identical TCR β-chains (including the CDR regions; not shown) are highlighted in the same colour. BV, β-chain variable region.
